# Hippocampal synaptic plasticity injury mediated by SIRT1 downregulation is involved in chronic pain‐related cognitive dysfunction

**DOI:** 10.1111/cns.14410

**Published:** 2023-08-17

**Authors:** Yanping Liu, Qiang Liu, Haibi Wang, Yongkang Qiu, Jiatao Lin, Weifeng Wu, Ning Wang, Wei Dong, Jie Wan, Chen Chen, Shuai Li, Hui Zheng, Yuqing Wu

**Affiliations:** ^1^ Jiangsu Province Key Laboratory of Anesthesiology/NMPA Key Laboratory for Research and Evaluation of Narcotic and Psychotropic Drugs Xuzhou Medical University Xuzhou China; ^2^ Department of Anesthesiology, National Cancer Center/National Clinical Research Center for Cancer/Cancer Hospital Chinese Academy of Medical Sciences and Peking Union Medical College Beijing China

**Keywords:** chronic pain, cognitive dysfunction, hippocampus, SIRT1, synaptic plasticity

## Abstract

**Aims:**

Cognitive dysfunction associated with chronic pain may be caused by impaired synaptic plasticity. Considering the impact of silent information regulator 1 (SIRT1) on synaptic plasticity, we explored the exact role of SIRT1 in cognitive impairment caused by chronic pain.

**Methods:**

We evaluated the memory ability of mice with the fear conditioning test (FCT) after spared nerve injury (SNI) model. Western blotting and immunofluorescence were used to analyze the expression levels of SIRT1. Hippocampal synaptic plasticity was detected with Golgi staining, transmission electron microscopy, and long‐term potentiation (LTP). In the intervention study, AAV9‐CaMKIIα‐Cre‐EGFP was injected to SIRT1^flox/flox^ mice to knockdown the expression levels of SIRT1. Besides, SNI mice were injected with AAV2/9‐CaMKIIα‐SIRT1‐3*Flag‐GFP or SRT1720 to increase the expression levels or enzymatic activity of SIRT1.

**Results:**

Our current results indicated that cognitive function in SNI mice was impaired, SIRT1 expression in glutaminergic neurons in the hippocampal CA1 area was downregulated, and synaptic plasticity was altered. Selective knockdown of SIRT1 in hippocampus damaged synaptic plasticity and cognitive function of healthy mice. In addition, the impaired synaptic plasticity and cognitive dysfunction of SNI mice could be improved by the upregulation of SIRT1 expression or enzyme activity.

**Conclusions:**

Reduced SIRT1 expression in hippocampus of SNI mice may induce cognitive impairment associated with chronic pain by mediating the impaired synaptic plasticity.

## Graphical abstract

The diagram illustrates the role of SIRT1 in the development of chronic pain‐related cognitive dysfunction. After nerve injury, the reduction in SIRT1 levels leads to fewer dendritic crossings, shorter overall dendritic length, decreased dendritic spine density, lower PSD95 levels, reduced thickness of the postsynaptic densities, widened synaptic fissure and lower fEPSP slope, ultimately leading to cognitive dysfunction.

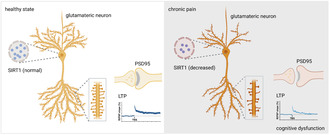

## 1 INTRODUCTION

According to epidemiological research, 20%–30% of people experience chronic pain,^1^, ^2^, ^3^, ^4^ which has a negative impact on people's social interaction, capacity for work, emotions, and quality of life.^5^, ^6^ The process by which the brain gathers, processes, stores, and retrieves information is referred to as cognition.^7^ Chronic pain is frequently linked to cognitive dysfunction, which can reduce attention span, psychomotor function, decision‐making skills, and execution capacity.^5^ However, the exact mechanism of cognitive dysfunction linked to chronic pain is still unknown.

Synaptic plasticity is the ability of neural networks in the brain to change through development and rearrangement and can be generally divided into structural plasticity and functional plasticity.^8^, ^9^, ^10^ Long‐term changes in synaptic function in selective brain circuits are considered the physical basis for memory storage, and activity‐dependent changes in synaptic connection strength are crucial for the formation and maintenance of memory.^11^ Compared with the synaptic plasticity of other brain regions, hippocampal synaptic plasticity is more vulnerable to the long‐term effects of stimulation.^12^ A large number of studies have shown that reduced cognitive performance may result from impaired synaptic plasticity in the hippocampal CA1 area.^13^, ^14^ Furthermore, hippocampal glutamatergic synapse impairment mediates novel‐object recognition dysfunction in rats with neuropathic pain.^15^, ^16^

Silent information regulator 1 (SIRT1), the human equivalent of Sir2 in yeast, is a histone deacetylase that is dependent on nicotinamide adenine dinucleotide (NAD+), and plays a key role in aging, metabolism, immunity, stress, and tumors. Recent studies have revealed that SIRT1 can lessen the cognitive impairment caused by Alzheimer's disease (AD).^17^, ^18^ A SIRT1‐specific agonist known as SRT1720 can treat cognitive abnormalities caused by hereditary cobalamin disease and RNA‐binding protein mislocalization.^19^ SIRT1 can regulate memory formation and synaptic plasticity via microRNA‐mediated pathways.^20^ However, the effect of SIRT1 in cognitive dysfunction impairment caused by chronic pain is uncertain.

Therefore, in this study, we explored the exact role of deacetylase SIRT1‐mediated synaptic plasticity of hippocampus in chronic pain‐related cognitive dysfunction and provided possible treatment strategies.

## 2 MATERIALS AND METHODS

### 2.1 Animals

The Xuzhou Medical University Animal Care and Use Committee has given its clearance to all practices involving animal care and treatment (ethical statement approval number: 202211S034). B6;129‐SIRT1tm1Ygu/J (SIRT1^flox/wt^) mice were purchased from the Jackson Laboratory. By inbreeding SIRT1^flox/wt^ mice, SIRT1^flox/flox^ mice were produced. C57BL/6J mice (8–12 weeks, 20–25 g) were purchased from Shandong Jinan Pengyue Experimental Animal Breeding Co., Ltd. All mice were housed in cages (3–5 mice/cage) with typical laboratory conditions (22 to 25°C, 40% to 60% humidity, 12 h of light and dark, free access to food and water).

### 2.2 SNI mouse model

**FIGURE 1:**
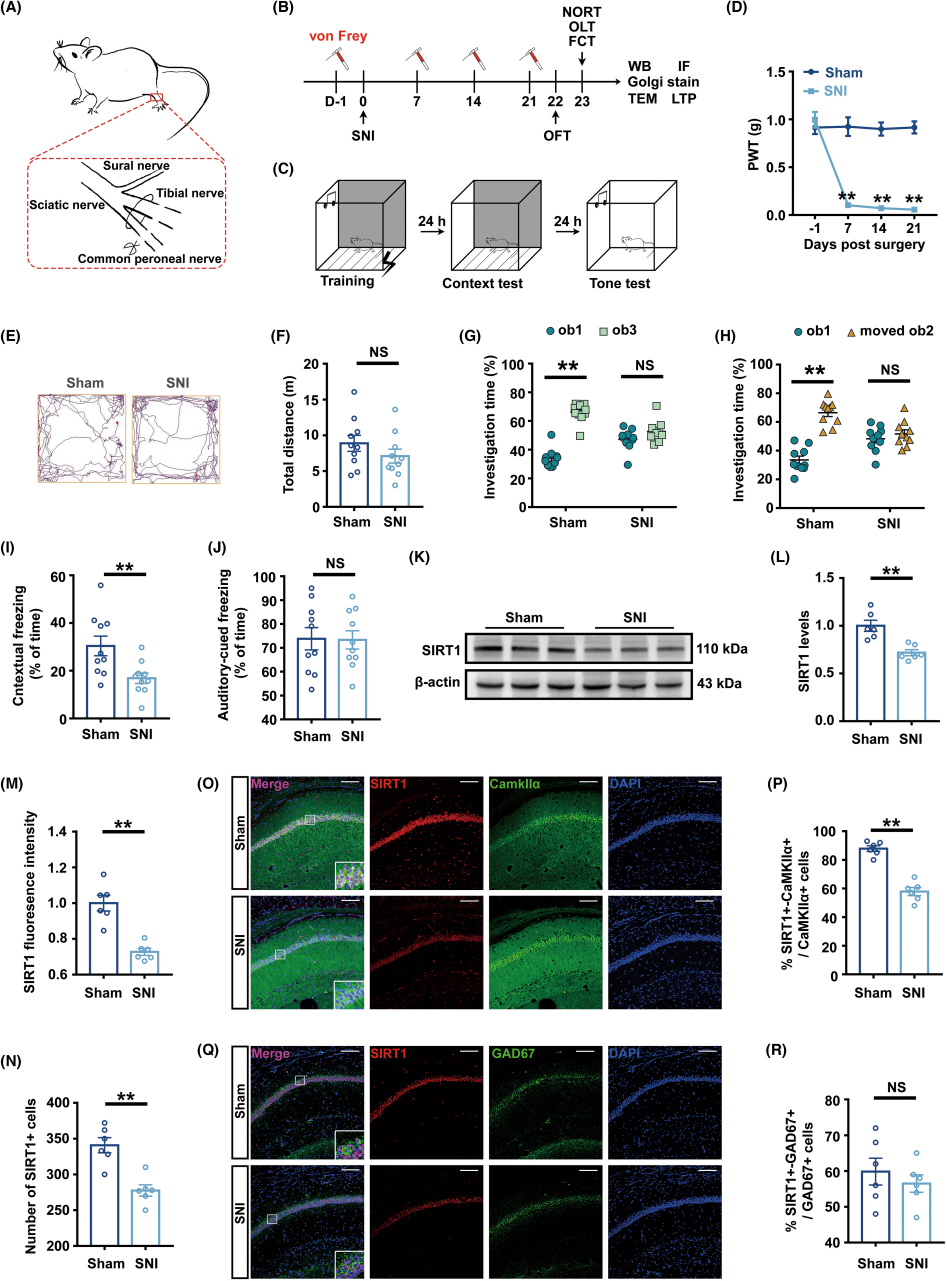
Chronic pain damaged hippocampus‐dependent cognitive function and downregulated SIRT1 expression in glutaminergic neurons of the hippocampal CA1 area. (A) Schematic diagram of SNI operation, including the transection of tibial and fibular branches of the sciatic nerve, preserving the integrity of the sural nerve. (B) A flow chart of the experiment. (C) Experiment diagram of FCT. (D) PWT of SNI and Sham mice 1 day before the operation and 7, 14, and 21 days after the operation (*n* = 10). **p < 0.01 versus the Sham group. (E) Representative track diagrams in the OFT. (F) The total movement distance in the OFT (*n* = 10). (G) Investigation time (%) to novel object in NORT test (*n* = 10). (H) Investigation time (%) to new location in OLT test (*n* = 10). (I) Freezing time in the context test (*n* = 10). (J) Freezing time in the tone test (*n* = 10). (K) Representative Western blot bands of SIRT1. (L) Quantitative results showing reduced SIRT1 protein levels in the hippocampus of SNI mice (*n* = 6). The expression of SIRT1 was normalized to that of β‐Actin in each sample. SIRT1 levels in the Sham group were set as 1 for quantification. (M) Quantitative results showing decreased overall fluorescence intensity of SIRT1 in SNI (*n* = 6). The fluorescence intensity of SIRT1 in the Sham group was set as 1 for quantification. (N) Quantitative results showing a decreased number of SIRT1^+^ cells in SNI (*n* = 6). (O) Representative colabeling images of SIRT1 and CaMKIIα. Scale bar, 100 μm. (P) Quantitative results showing a decreased colabeling rate of SIRT1 and CaMKIIα in SNI (*n* = 6). (Q) Representative colabeling images of SIRT1 and GAD67. Scale bar, 100 μm. (R) There was no difference in the colabeling rates of SIRT1 and GAD67 between the Sham and SNI groups (*n* = 6). **p < 0.01; NS, no significance. Error bars indicate SEM. SNI, spared nerve injury; OFT, open field test; FCT, fear conditioning test; PWT, paw withdrawal threshold; SIRT1, silent information regulator 1.

The left tibial and common peroneal nerve branches of the sciatic nerve in mice were ligated and severed according to the spared nerve injury (SNI) model previously performed.^21^ Briefly, under the anesthesia with sevoflurane inhalation, the common peroneal nerve and tibial nerve of left thigh were tied off with 4.0 silk thread, and the distal of the ligations was sectioned, without the sural nerve being stimulated during the procedure (Figure 1A). The sciatic nerve and its branches were solely exposed to the sham mice, but no branches were cut.

### 2.3 AAV vectors and viral microinjections

The viral vectors AAV9‐CaMKIIα‐Cre‐EGFP (AAV‐Cre) and AAV9‐CaMKIIα‐EGFP (AAV‐EGFP), AAV2/9‐CaMKIIα‐SIRT1‐3*Flag‐GFP (AAV‐SIRT1), and AAV2/9‐CaMKIIα‐GFP (AAV‐GFP) were obtained from BrainVTA Co., Ltd. and Hanbio Co., Ltd., respectively. Stereotaxic surgeries were carried out under the anesthesia with inhalation of sevoflurane combined with intraperitoneal injection of chloral hydrate to microinject the viral vectors bilaterally (0.2 μL on each side) into the hippocampal CA1 area (AP = −1.95 mm, ML = ± 1.4 mm, DV = −1.4 mm from the bregma) at a rate of 0.05 μL/min. The GFP or EGFP fluorescence signal in the hippocampal brain tissue sections confirmed the site of virus transfection.

### 2.4 Catheterization and agonist injection

Under the anesthesia with inhalation of sevoflurane combined with intraperitoneal injection of chloral hydrate, stereotactic administration was performed by placing the double catheter (Rwd Life Science Co., Ltd) on the 11th day after SNI surgery and SRT1720 (MedChemExpress, 200 nL every day on each side, 100 nL/min, 0.8 μg/10 μL) was delivered into the hippocampal CA1 area (AP = −1.95 mm, ML = ±1.4 mm, DV = −1.4 mm from the bregma) on the 18th, 19th, 20th, and 21st day after SNI surgery. To ensure proper healing, catheters were implanted 1 week before administration. The needle was kept in place for 10 min after the injection, and then it was removed and an internal core was inserted to seal the hole and avoid clogging.

### 2.5 Behavioral tests

#### 2.5.1 Paw withdrawal threshold

All behavioral investigations were carried out by an investigator blinded to the treatment group. Before testing, mice were kept in test chambers (8 × 8 × 5.5 cm^3^, LWH) with a bottom of wire mesh for a 30‐min acclimatization period. To quantify mechanical allodynia, a series of ascending force von Frey monofilaments (0.008, 0.02, 0.07, 0.16, 0.4, 1, 2, 6 g, North Coast Medical) were placed on the left hind paw's lateral plantar surface. To estimate 50% paw withdrawal threshold (PWT), a simplified up‐and‐down method was employed referring to the previous study.^22^ Briefly, starting with the 0.16‐g filament, use the subsequent lower‐weight filament with a 5‐min gap between each stimulus if the withdrawal is noticed within 5 s; otherwise, use the heavier filament. After the withdrawal was noticed, continue to record responses for four filaments. The 50% PWT was converted as previously described.^23^

#### 2.5.2 Open field test

The locomotor activity of mice was assessed using an open field test (OFT). Mice were placed separately in a square field (50 × 50 × 50 cm^3^) to wander around freely for 5 min after adapting the room for 1 h. The interior of the box was wiped with 75% ethanol to remove the last mouse's odor. The movement traces of the mice were tracked and recorded using the ANY‐maze software system (Stoelting Co.) with the total distance calculated.

#### 2.5.3 Novel object recognition test (NORT)

In accordance with earlier research,^24^, ^25^, ^26^ NORT was carried out in the same cube as OFT. Mice were given 10 min to become accustomed to their surroundings. After a period of 24 h, the mice were exposed for 10 minutes to the cube containing two objects (ob1 and ob2), and the amount of time they spent in investigating each object was noted. The mice were put back in the cube after 2 h, but ob2 had been switched out for ob3. Keep track of the time spent in investigating both ob1 and ob3 (Figure S1A). The percentage of time spent in exploring one object relative to the sum of all time spent in exploring the two objects was used to calculate the investigating time (%). The proportion of time spent in exploring ob3 showed a preference for novel things.

#### 2.5.4 Object location test (OLT)

OLT was carried out in the same cube as NORT. Mice were given 10 min to become accustomed to their surroundings. After a period of 24 h, the mice were exposed for 10 minutes to the cube containing two objects (ob1 and ob2), and the amount of time they spent in investigating each object was noted. The mice were put back in the cube after 2 h, but ob2 was moved to a new corner. Keep track of the time spent in investigating both ob1 and moved ob2 (Figure S1B). The percentage of time spent in exploring one object relative to the sum of all time spent in exploring the two objects was used to calculate the investigating time (%). The proportion of time spent in exploring moved ob2 showed a preference for new location.

#### 2.5.5 Fear conditioning test

Refer to previous research,^27^ the mice were given 2 min to adjust in a box with a fence at the bottom and a black wall all around during the training (defined as Base). three pairs of conditional (20 s, 4 kHz tone, 60 s interval) and unconditional (2 s at the end of every tone, 0.7 mA) stimuli were exposed to the mice (defined as S1, S2, S3). System software (Med Associates, Inc.) was used to record the freezing times in the stage of base, S1, S2, and S3. The mice were placed in the same box at the training stage for 8 min after 24 h with the percentage of freezing time noted. After another 24 h, the mice were placed in another box with whiteboards at the bottom and around it and received a conditional stimulation (5 min, 4 kHz tone) with the percentage of the freezing time recorded (Figure 1C). The context test was used to evaluate hippocampus‐dependent memory (contextual fear memory), while the tone test was used to evaluate hippocampus‐independent memory (auditory‐cued fear memory).^14^, ^27^

### 2.6 Western blotting

RIPA lysis buffer (Beyotime) containing PMSF was used to homogenize the hippocampal tissue. The protein concentrations were measured using a BCA kit (Beyotime) and trimmed with RIPA lysis buffer. Following a 10‐min boiling, the samples were kept at −20°C. The proteins were separated using 12.5% gradient sodium dodecyl sulfate‐polyacrylamide electrophoresis (SDS–PAGE) and were then transferred to PVDF membranes (Merck Millipore, ISEQ00010). After being blocked with 5% nonfat milk for 2 h, the membranes were incubated with primary antibodies overnight at 4°C, following by incubation with horseradish peroxidase binding antibodies (1:2000, Beyotime) for 1 h at room temperature. Primary antibodies included anti‐SIRT1 (1:1000, 9475S, Cell Signaling Technology), anti‐PSD95 (1:1000, 2507S, Cell Signaling Technology), and anti‐β‐actin (1:2000, AC004, ABclonal, China). Protein bands of the membranes were visualized using ECL detection equipment (Beyotime) and quantified with ImageJ software.

### 2.7 Immunofluorescence

After mice were perfused with 0.9% saline following by 4% paraformaldehyde under sevoflurane anesthesia, the mice brains were harvested, fixed in 4% paraformaldehyde for 6–8 h, and dehydrated in 30% sucrose for 3 days. Using a frozen microtome (CM1950, Leica), the mice brains were cut into 30‐μm‐thick coronal sections. After antigen retrieval, the brain sections were ruptured of membranes with 0.8% PBST and then blocked with 5% goat serum. The brain sections were incubated with primary antibodies for 3 days and nights at 4°C. We used the following primary antibodies for immunofluorescence: anti‐SIRT1 (1:100, 9475S, Cell Signaling Technology) mixed with anti‐GAD67 (1:300, ab26116, Abcam) and anti‐SIRT1 mixed with anti‐CamKIIα (1:300, 50049S, Cell Signaling Technology). Thereafter, the brain sections were probed with fluorescent secondary antibodies (1:400, ab150080, ab150113, ab150115, Abcam) for 1 h at 37°C. The nucleus was stained with DAPI (ab104139, Abcam). A confocal microscope was used to view the staining outcomes (FV1000, Olympus).

### 2.8 Golgi‐cox staining

Following the FD Fast Golgi Staining Kit's (FD, PK401A) instructions, the brain tissue was immersed in mixed solution of liquid A and B for 14 days at room temperature. After transferred into liquid C to be dehydrated for 3 days, the brain tissue was cut into coronal sections (120 μm) using a vibrating microtome (VT1000S, Leica Microsystems). Gelatin‐coated microscope slides were used to load the brain sections, which were then dyed for 10 min with a mixture of liquid D and E. After gradient alcohol dehydration and xylene transparency, the brain sections were sealed with a neutral resin. The entire procedure was performed in a dim environment. Dendrites and dendritic spines of neurons in the hippocampal CA1 area were imaged with an Olympus BX53 microscope and analyzed with ImageJ software. Eighteen neurons were counted for statistical analysis. Specifically, three animals in each group were utilized for Golgi‐Cox staining, and two neurons in the CA1 region of each coronal slice at the anterior, middle, and posterior positions of a mouse were selected for analysis.

### 2.9 Transmission electron microscopy (TEM)

Hippocampal tissue (1 × 1 × 1 mm^3^) harvested was prefixation with a mixture of 2.5% glutaraldehyde and 2% paraformaldehyde for more than 24 h. Then, the brain tissue was fixed with 1% osmic acid for 2 h. After undergoing gradient dehydration with ethanol and acetone, the brain tissue was submerged and implanted in Epon resin. After that, an ultrathin slicer ((UC7rt) A‐1170) was used to cut the tissue into ultrathin sections (70 nm thick), which collected on copper mesh and stained with uranium acetate and lead citrate. Photographs of the asymmetric synapses that mediate excitatory conduction were taken using transmission electron microscopy (Tecnai G2S pirit Twin).^28^ Under a 5800× microscope, two images of one ultrathin segment from three mice, respectively in each group were captured. The thickness of the postsynaptic dense substance (PSD) and the synaptic space (SC) of a synapse were measured separately as the average length of the vertical lines from the postsynaptic membrane to the synaptic complex and from the presynaptic membrane to the postsynaptic membrane at five different locations by ImageJ software. The average thickness of the PSD or SC across all synapses in an image was then used as a single piece of data.

### 2.10 Long‐term potentiation (LTP) recording

Referring to the previous literature,^29^ after mice were perfused with frozen cutting solution preoxygenated with 95% oxygen and 5% carbon dioxide under sevoflurane anesthesia, the mice brains were quickly extracted and cut into hippocampal coronal sections (300 μm thick) in cutting solution using a concussion slicer. The brain sections were incubated in oxygenated artificial cerebrospinal fluid (ACSF) for 1 h at 32°C. Then the Schaffer collateral branches were stimulated with electrodes and field excitatory postsynaptic potential (fEPSP) of CA1 area were recorded using a glass microtubule pipette filled with ACSF. Three trains of theta burst stimulation (TBS; duration 1 s at 100 Hz with 20‐s interval for each train) were delivered to the slice to induce long‐term potentiation (LTP). The fEPSP with stable slope was captured for 0.5 h before TBS (recorded as baseline 1). After TBS, the changes in fEPSP and its slope were monitored for an additional 1 h (recorded as waveform 2) and the average slopes during the last 20 min were analyzed.

### 2.11 Nissl staining

The site of catheterization was determined by Nissl staining. After prepared with the same technique in the aforementioned immunofluorescence experiment, the brain sections were stained using Nissl staining solution (Beyotime) for 10 min at 40°C, following by being rinsed in distilled water, dehydrated in 95% ethanol, cleared in xylene, and sealed with neutral resin. The outcome was observed using an Olympus BX53 microscope.

### 2.12 Statistical analysis

The data were analyzed using GraphPad Prism 7.0 (GraphPad Software, Inc.). All data followed a normal variable distribution, which was checked by the Shapiro–Wilk test. The results are shown as the mean ± SEM. The unpaired t test was used to compare the differences between the two groups. To calculate the differences among the four groups, one‐way ANOVA was performed. Two‐way ANOVA followed by Tukey's multiple‐comparison test was used to compare how two factors affected a numeric result. The threshold for significance was set at *p* < 0.05.

## 3 RESULTS

### 3.1 Chronic pain induced cognitive dysfunction in mice

Prior research has shown that 21 days after modeling, animals with persistent neuropathic pain displayed cognitive impairment.^30^ Figure 1B showed a flow chart of the experiment and Figure 1C showed flow chart of FCT. In our study, compared to Sham mice, the PWT in SNI mice showed no difference before surgery but dropped on the 7th, 14th, and 21st days after surgery (Figure 1D). When we evaluated the mice's ability to exercise following surgery on Day 22, SNI mice showed no difference in a total exercise distance compared to Sham mice (Figure 1E,F). Utilizing mice's natural curiosity about new items, NORT was used to test their recognition and memory abilities. Mice's capacity for spatial memory was further examined by OLT. The findings demonstrated that there was no significant difference between the two groups in mice's exploration time percentage toward the two objects during the training phase (Figure S1C,D). In the testing phase, sham mice spent more time exploring ob3 or moved ob2, whereas SNI mice spent about the same amount of time exploring both ob1 and ob3 in NORT or ob1 and moved ob2 in OLT (Figure 1G,H). Hippocampus‐dependent memory was assessed through the context test in the FCT, whereas hippocampus‐independent memory was assessed via the tone test.^31^ There was no discernible difference between the two groups on the freezing time in the fear conditioning training stage or the FCT tone test (Figure S1E, Figure 1J). However, compared with that of Sham mice, the freezing time of SNI mice during the FCT context test was dramatically reduced (Figure 1I).

### 3.2 SIRT1 expression in glutaminergic neurons of the hippocampal CA1 area was downregulated in SNI mice

Hippocampal SIRT1 plays a critical role in adult learning and memory development.^17^, ^18^, ^19^, ^20^ The results of Western blotting revealed that the hippocampus of SNI mice contained lower SIRT1 expressions (Figure 1L). Besides, the immunofluorescence outcomes demonstrated a decrease in the overall fluorescence intensity of SIRT1 and the number of SIRT1^+^ cells in the hippocampus of SNI mice (Figure 1M,N). The colabeling rate of SIRT1 with glutaminergic neurons or GABA neurons was analyzed to explore the neuron types of these SIRT1 changes. The results showed that compared with Sham mice, SIRT1 colabeling rates with glutaminergic neurons in SNI mice were lower but these with GABAergic neurons were not significantly altered (Figure 1O–R).

### 3.3 Chronic pain impaired structural and functional synaptic plasticity of the hippocampus

**FIGURE 2:**
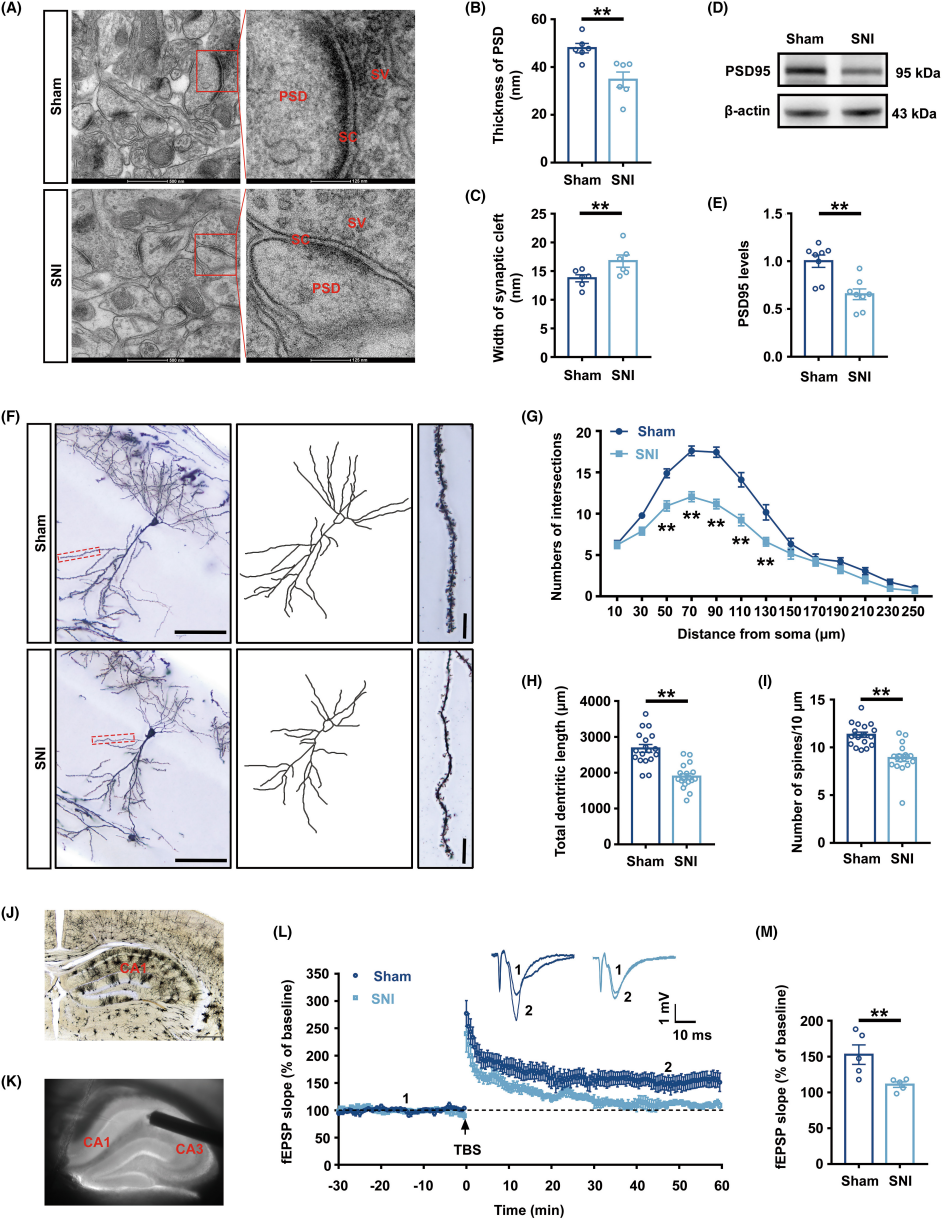
Structural and functional synaptic plasticity of the hippocampal CA1 area was impaired in SNI mice. (A) The ultrastructure of synapses on the electron micrograph in the hippocampus. (B, C) Image analysis of the thickness of PSD and the width of the synaptic cleft (*n* = 6). (D) Representative Western blot bands of PSD95. (E) Decreased PSD95 protein levels in the hippocampus of SNI mice (*n* = 6). The expression of PSD95 was normalized to that of β‐Actin for each sample. PSD95 levels in the Sham group were set as 1 for quantification. (F) A hippocampal profile image of Golgi staining of hippocampal CA1 neurons, 20× with camera tracings and 60× for spine counting. Scale bar, 100 μm for 20×; 10 μm for 60×. (G) Quantitation of dendritic intersections (*n* = 18). **p < 0.01 versus the Sham group. (H) Quantitation of the total dendritic length (*n* = 18). (I) Quantitation of the dendritic spine density (*n* = 18). (J) A hippocampal profile image of Golgi staining of 4×. Scale bar, 500 μm. (K) Sample image showing the location of stimulation in the Schaffer collateral and recording in the CA1 region. (L) fEPSP slope before and after TBS was recorded as baseline 1 (before TBS) and representative waveform 2 (after TBS). The arrow indicated the time point of TBS application. (M) The average fEPSP slope at 40–60 min after TBS (*n* = 5). **p < 0.01; Error bars indicate SEM. PSD95, postsynaptic density 95; SNI, spared nerve injury; LTP, long‐term potentiation; TBS, theta burst stimulation; fEPSP, field excitatory postsynaptic potential.

Which the structural plasticity of hippocampal neurons is crucial for learning and memory has been confirmed in previous studies.^14^, ^32^ TEM was used to investigate alterations in the synaptic structure of the hippocampal CA1 area in SNI mice. The presynaptic end was somewhat enlarged, and the thickness of the postsynaptic densities was reduced as the synaptic fissure widened in SNI mice (Figure 2A–C). Furthermore, the expression of postsynaptic density 95 (PSD95), a crucial postsynaptic protein, was assessed by Western blotting. The SNI group had a lower expression of PSD95 protein in comparison to that in the Sham group (Figure 2D,E). To study the consequences of persistent pain on the dendrites and dendritic spines of neurons, Golgi‐Cox staining was utilized. The graphics displayed the 4×, 20×, and 60× scopes of neurons in the CA1 of Sham and SNI (Figure 2F,J). Sholl analysis was used to quantify the dendritic branches and overall dendritic length. In comparison to the Sham group, the SNI group had fewer dendritic crossings (between 50 and 130 μm from the neuronal soma center) and a shorter overall dendritic length (Figure 2G,H). A decrease in dendritic spine density was also observed in SNI mice, according to a study of dendritic spine density at 60× magnification (Figure 2I).

LTP, a studied model of synaptic plasticity that has received considerable attention, was first discovered in the hippocampus.^33^ To learn about the effect of chronic pain on functional synaptic plasticity, we used the principle of electrophysiological LTP to record the fEPSP induced by Schaffer collateral between CA3 and CA1 in hippocampal sections from Sham and SNI mice (Figure 2K). The results indicated that SNI mice sections exhibited lower levels of fEPSP slopes after TBS (Figure 2L,M).

### 3.4 Specific knockdown of SIRT1 in glutaminergic neurons of the hippocampal CA1 area impaired hippocampal synaptic plasticity and cognitive dysfunction

**FIGURE 3:**
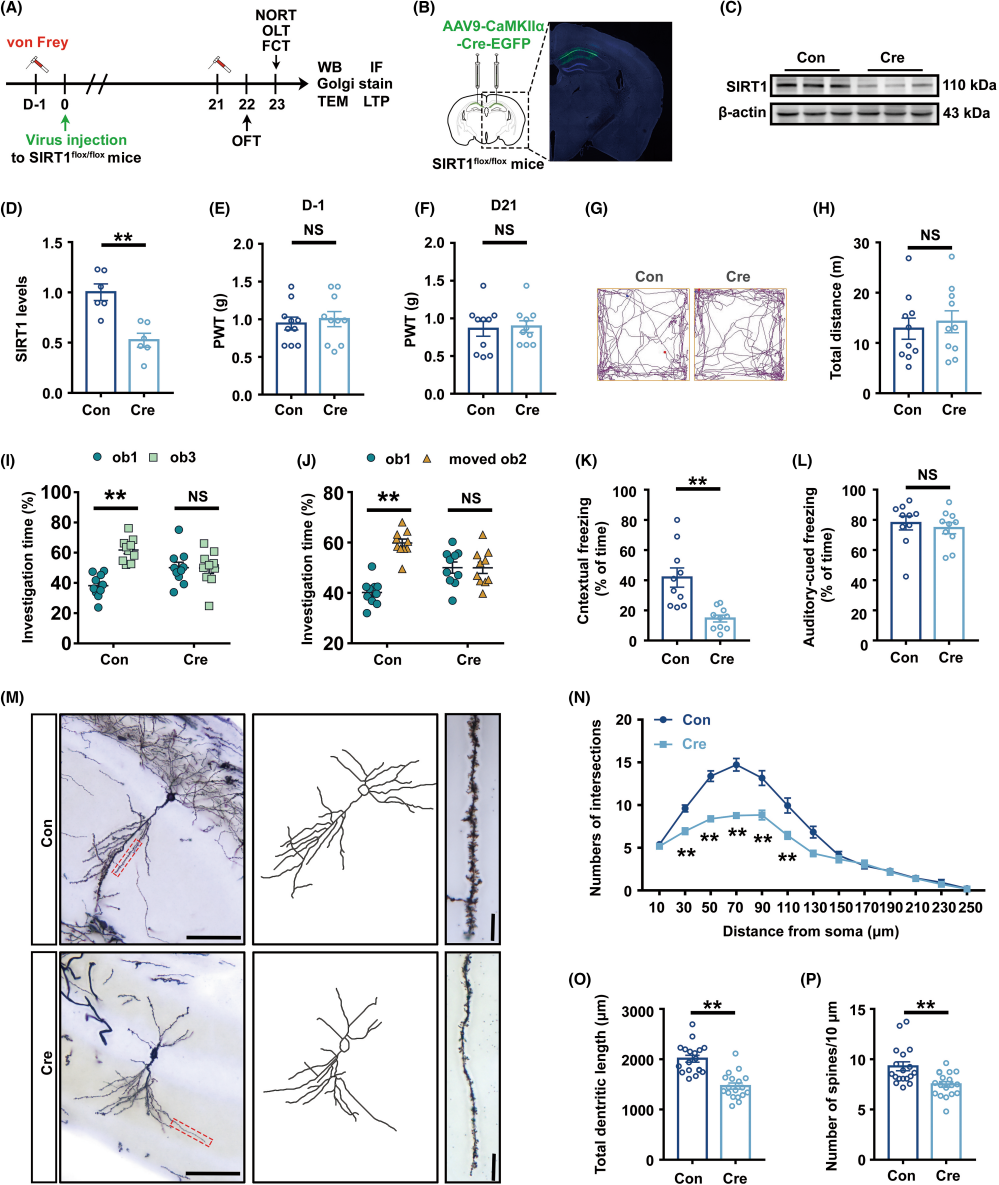
Hippocampal synaptic plasticity and cognitive dysfunction were specifically affected by SIRT1 knockdown in glutaminergic neurons of the hippocampal CA1 area. (A) A flow chart of the experiment. (B) Fluorescence images showing efficient expression of the AAV‐Cre vector in the CA1 region. (C) Representative Western blot bands of SIRT1. (D) Quantitative results showing that AAV‐Cre injection downregulated SIRT1 expression in the hippocampus (*n* = 6). The expression of SIRT1 was normalized to that of β‐Actin for each sample. SIRT1 levels in the Con group were set as 1 for quantification. (E) PWT of Con and Cre mice 1 day before virus injection (*n* = 10). (F) PWT of Con and Cre mice 21 days after virus injection (*n* = 10) (G) Representative track diagrams of Con and Cre mice in the OFT. (H) The total movement distance of Con and Cre mice in the OFT (*n* = 10). (I) Investigation time (%) to novel object in NORT test (*n* = 10). (J) Investigation time (%) to new location in OLT test (*n* = 10). (K) Freezing time in the context test (*n* = 10). (L) Freezing time in the tone test (*n* = 10). (M) A hippocampal profile image of Golgi staining of hippocampal CA1 neurons, 20× with camera tracings and 60× for spine counting. Scale bar, 100 μm for 20×; 10 μm for 60×. (N) Quantitation of dendritic intersections (*n* = 18). **p < 0.01 versus the Sham group. (O) Quantitation of the total dendritic length (*n* = 18). (P) Quantitation of the dendritic spine density (*n* = 18). **p < 0.01; NS, no significance. Error bars indicate SEM. SIRT1, silent information regulator 1; OFT, open field test; FCT, fear conditioning test; PWT, paw withdrawal threshold.

To probe whether SIRT1 in hippocampal glutaminergic neurons is the key to the development of cognitive dysfunction, the AAV‐Cre vector was injected into the hippocampal CA1 area of SIRT1^flox/flox^ mice, and the AAV‐GFP vector was used as a control. Figure 3A showed a flow chart of the experiment. The fluorescent representative image of the virus showed that the virus was limited to the hippocampal CA1 area (Figure 3B). Western blotting analysis showed that SIRT1 expression in the hippocampus of knockdown mice was considerably lower than that of the control group. (Figure 3C,D). One day before and 21 days after virus injection, knockdown and control mice displayed equivalent mechanical pain thresholds, demonstrating that knockdown of SIRT1 in glutaminergic neurons in the hippocampal CA1 area had no impact on mice's mechanical pain threshold (Figure 3E,F). Twenty‐two days after viral injection, knockdown mice's total exercise distance in OFT did not dramatically differ from control mice, indicating that the exercise capacity of mice is unaffected by virus injection (Figure 3G,H). During NORT or OLT training phase, the control and knockdown mice's investigation time toward ob1 and ob2 showed no significant difference (Figure S2A,B). In the testing phase, the SIRT1‐knockdown mice did not spend more time exploring ob3 or moved ob2 when compared to ob1 (Figure 3I,J). There was no discernible difference in the freezing time between the two groups during the FCT training stage and in the hippocampus‐independent tone test (Figure S2C, Figure 3L). However, knockdown mice had considerably shorter freezing times in the hippocampus‐dependent context test (Figure 3K).

Sholl analysis revealed that compared to control mice, knockdown mice had fewer dendritic crossings at 30–110 μm from the center of the cell body, and the total length of dendrites dropped (Figure 3M–O). Under a 60× microscope, neurons from knockdown mice had considerably less dendritic spine density (Figure 3M,P). In addition, compared to those in control mice, TEM results showed that the synaptic gap was significantly widened and the thickness of postsynaptic dense matter (PSD) was significantly reduced in SIRT1 knockdown mice (Figure S2D–F); The expression of PSD95 in the hippocampus was markedly reduced (Figure S2G,H); The LTP results showed that the average fEPSP slope was decreased significantly in the knockdown mice slices at 40–60 min after TBS (Figure S2I,J).

### 3.5 SRT1720 improved hippocampal cognitive dysfunction and synaptic plasticity impairment induced by chronic pain

**FIGURE 4:**
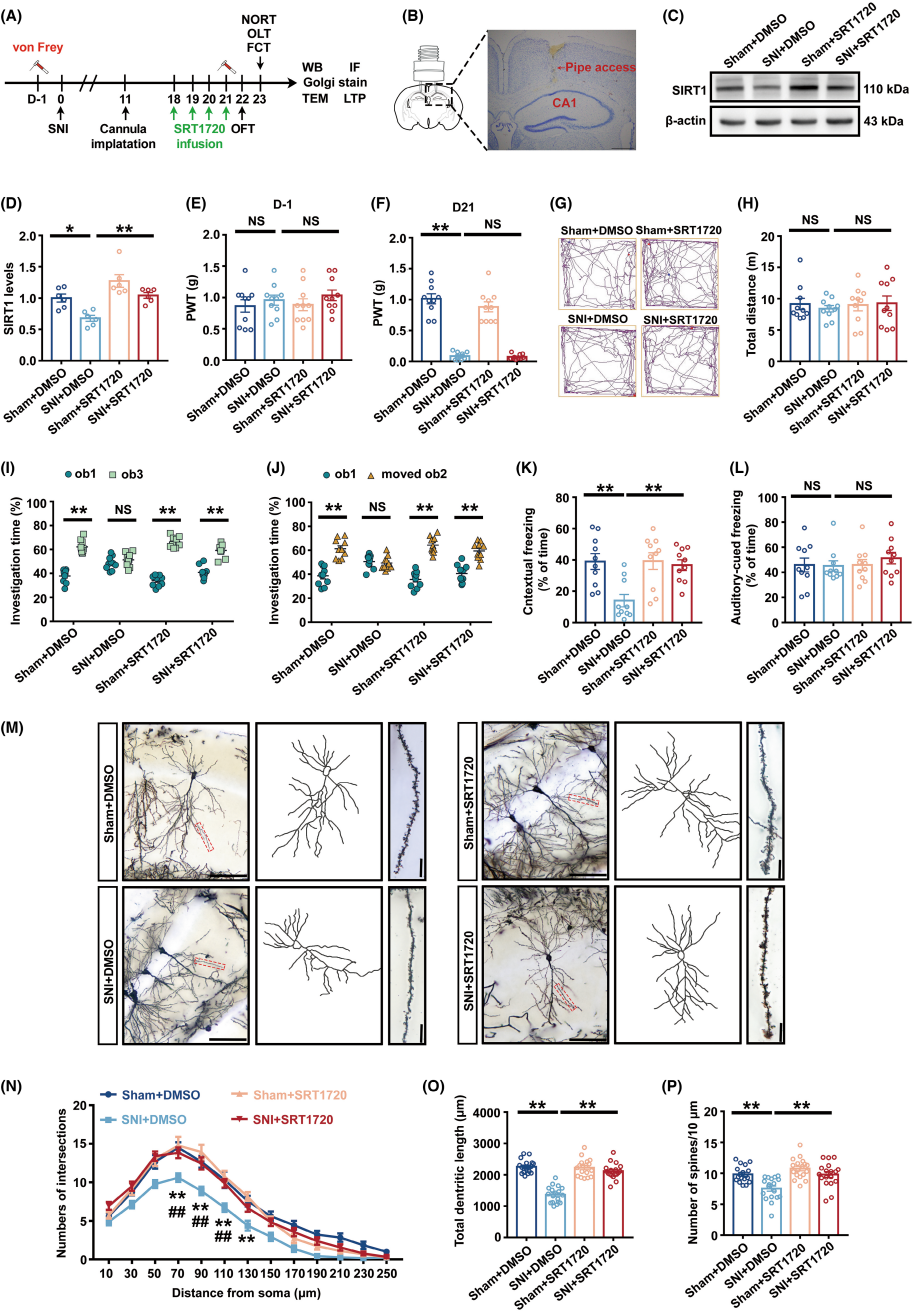
SRT1720 Improved cognitive dysfunction and hippocampal synaptic plasticity impairment caused by SNI modeling. (A) A flow chart of the experiment. (B) Nissl staining image showing that the cannula was implanted into the CA1 region. (C) Representative Western blot bands of SIRT1. (D) Quantification of SIRT1 expression (*n* = 6). The expression of SIRT1 was normalized to that of β‐Actin for each sample. SIRT1 levels in the Sham+DMSO group were set as 1 for quantification. (E) PWT 1 day before the operation (*n* = 10). (F) PWT on the 21st day after the operation (*n* = 10). (G) Representative track diagrams of the four groups in the OFT. (H) The total movement distance of the four groups in the OFT (*n* = 10). (I) Investigation time (%) to novel object in NORT test (*n* = 10). (J) Investigation time (%) to new location in OLT test (*n* = 10). (K) Freezing time in the context test. (*n* = 10). (L) Freezing time in the tone test (*n* = 10). (M) A hippocampal profile image of Golgi staining of hippocampal CA1 neurons, 20× with camera tracings, and 60× for spine counting. Scale bar, 100 μm for 20×; 10 μm for 60×. (N) Quantitation of dendritic intersections (*n* = 18). **p < 0.01 versus the Sham+DMSO group; ^##^ *p* < 0.01 versus the SNI + SRT1720 group. (O) Quantitation of the total dendritic length (*n* = 18). (P) Quantitation of the dendritic spine density (*n* = 18). **p < 0.01; *p < 0.05; NS, no significance. Error bars indicate SEM. SNI, spared nerve injury; OFT, open field test; FCT, fear conditioning test; PWT, paw withdrawal threshold.

To explore whether increasing the enzyme activity of SIRT1 could improve the impairment of cognitive dysfunction caused by SNI, we inserted a catheter into the hippocampal CA1 area of the mouse on the 11th day, and injected the SIRT1 agonist SRT1720 on the 18th, 19th, 20th, and 21st days, with DMSO as the control medium (Figure 4A). According to Nissl staining, the catheter entered the CA1 but did not injure pyramidal cells (Figure 4B). Western blot analysis showed that SNI+DMSO mice had much lower SIRT1 expression than Sham+DMSO mice, but SNI+SRT1720 animals had significantly higher SIRT1 expression. The findings demonstrated that SRT1720 counteracted the reduction in SIRT1 expression levels induced by SNI (Figure 4C,D). The outcomes in PWT demonstrated that there was no difference between the mechanical pain thresholds of the four groups of mice before modeling (Figure 4E). SNI+DMSO mice had a lower threshold for paw withdrawal than Sham+DMSO mice. SNI+DMSO and SNI+SRT1720 mice did not exhibit any noticeable differences in PWT (Figure 4F). There was no distinguishable difference between the four groups of mice's total distance in OFT, demonstrating that these models and agonists had no impact on the mice's capacity for exercise (Figure 4G,H). In the NORT and OLT studies, compared to familiar ob1, the SNI+DMSO group mice did not show more time investigating unfamiliar ob3 or move ob2, but the SNI+SRT1720 group mice significantly increased their exploration duration for unfamiliar ob3 and moved ob2 (Figure S3A,B and Figure 4I,J). In FCT, SNI+DMSO mice displayed a shorter freezing time than Sham+DMSO mice in the context test, while SNI+SRT1720 mice displayed a considerably longer freezing time than SNI+DMSO mice (Figure 4K). Additionally, in the FCT training phase and the hippocampal‐independent tone test, no significant difference was observed in freezing time among the four groups (Figure S3C, Figure 4L). The above results showed that SRT1720 prevented the cognitive dysfunction induced by SNI.

According to the findings of the Sholl analysis in Golgi staining, neurons of SNI+DMSO mice exhibited lower overall dendritic length and fewer dendritic crossings at 70–130 μm from the center of the cell body than Sham+DMSO mice. Compared to SNI+DMSO mice, SRT1720 dramatically increased frequency of dendritic crossings at 70–110 μm from the center of the cell body, total dendritic length of neurons, and the density of dendritic spines in SNI+SRT1720 mice (Figure 4M–P). It was also found that in the mice of SNI+SRT1720 group the synaptic gap was greatly reduced and the thickness of PSD was obviously increased when compared to those in the SNI+DMSO group mice detected by TEM (Figure S3D–F). In addition, the expression of hippocampal PSD95 was significantly elevated (Figure S3G,H) and the average fEPSP slope was significantly increased (Figure S3I,J) in SNI mice after SRT1720 administration. All the aforementioned findings indicated that enhancing SIRT1 enzyme activity by SRT1720 could mitigate the damage that SNI caused to synaptic plasticity.

### 3.6 Cognitive dysfunction and hippocampal synaptic plasticity injury induced by chronic pain were improved by upregulating SIRT1 expression in glutaminergic neurons

**FIGURE 5:**
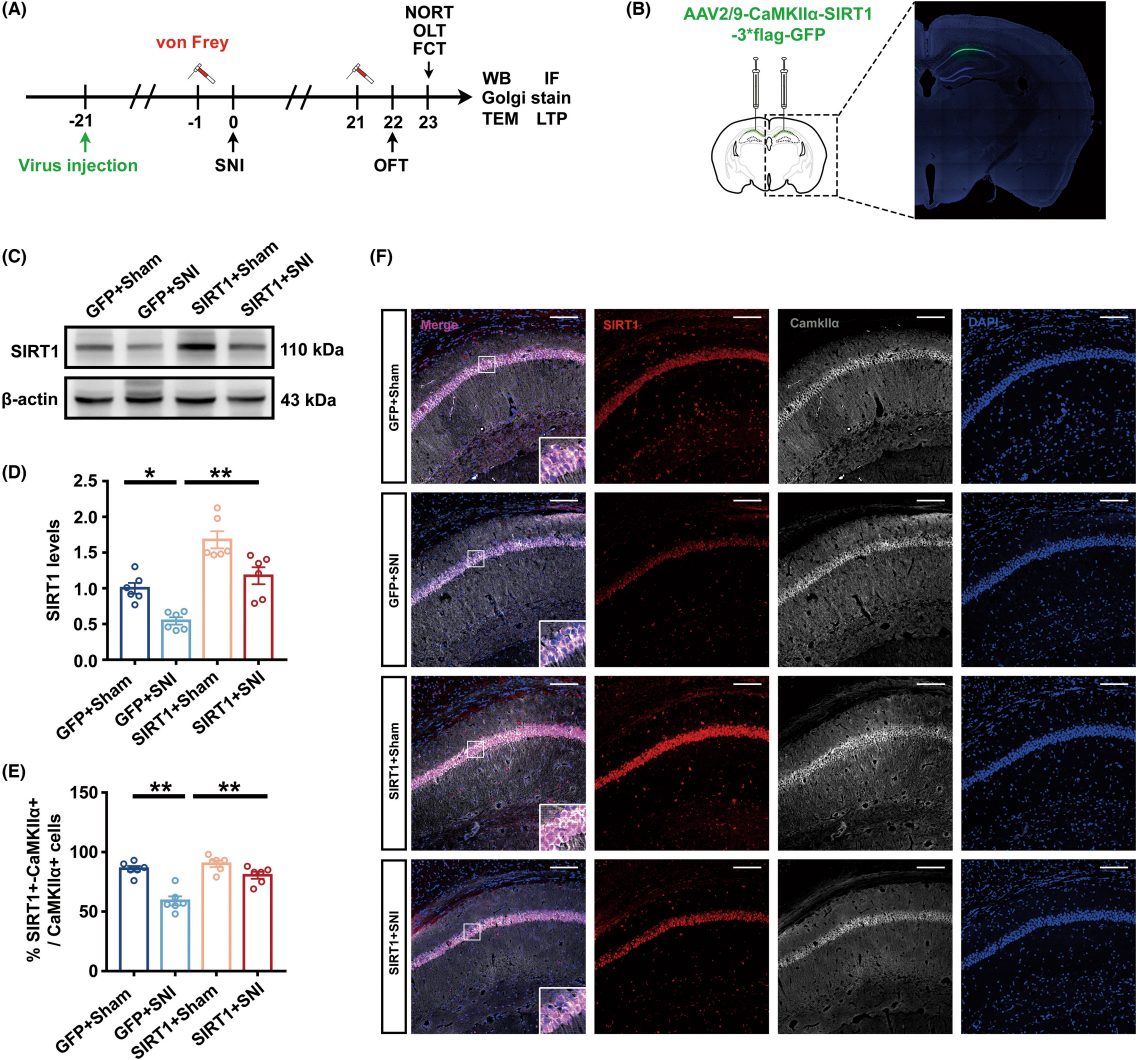
AAV‐SIRT1 upregulated the expression of SIRT1 in glutamatergic neurons of the hippocampal CA1 area of SNI mice. (A) A flow chart of the experiment. (B) Fluorescence images showing efficient expression of the AAV‐SIRT1 vector in the CA1 region. (C) Representative Western blot bands of SIRT1. (D) Quantitative results showing that AAV‐SIRT1 injection upregulated SIRT1 expression in the hippocampus of SNI mice (*n* = 6). The expression of SIRT1 was normalized to that of β‐Actin for each sample. SIRT1 levels in the GFP + Sham group were set as 1 for quantification. (E) Quantitation of the colabeling rate of SIRT1 and CaMKIIα (*n* = 6). (F) Representative colabeling images of SIRT1 and CaMKIIα. Scale bar, 100 μm. **p < 0.01; *p < 0.05. Error bars indicate SEM. SNI, spared nerve injuery; OFT, open field test; FCT, fear conditioning test; SIRT1, silent information regulator 1.

**FIGURE 6:**
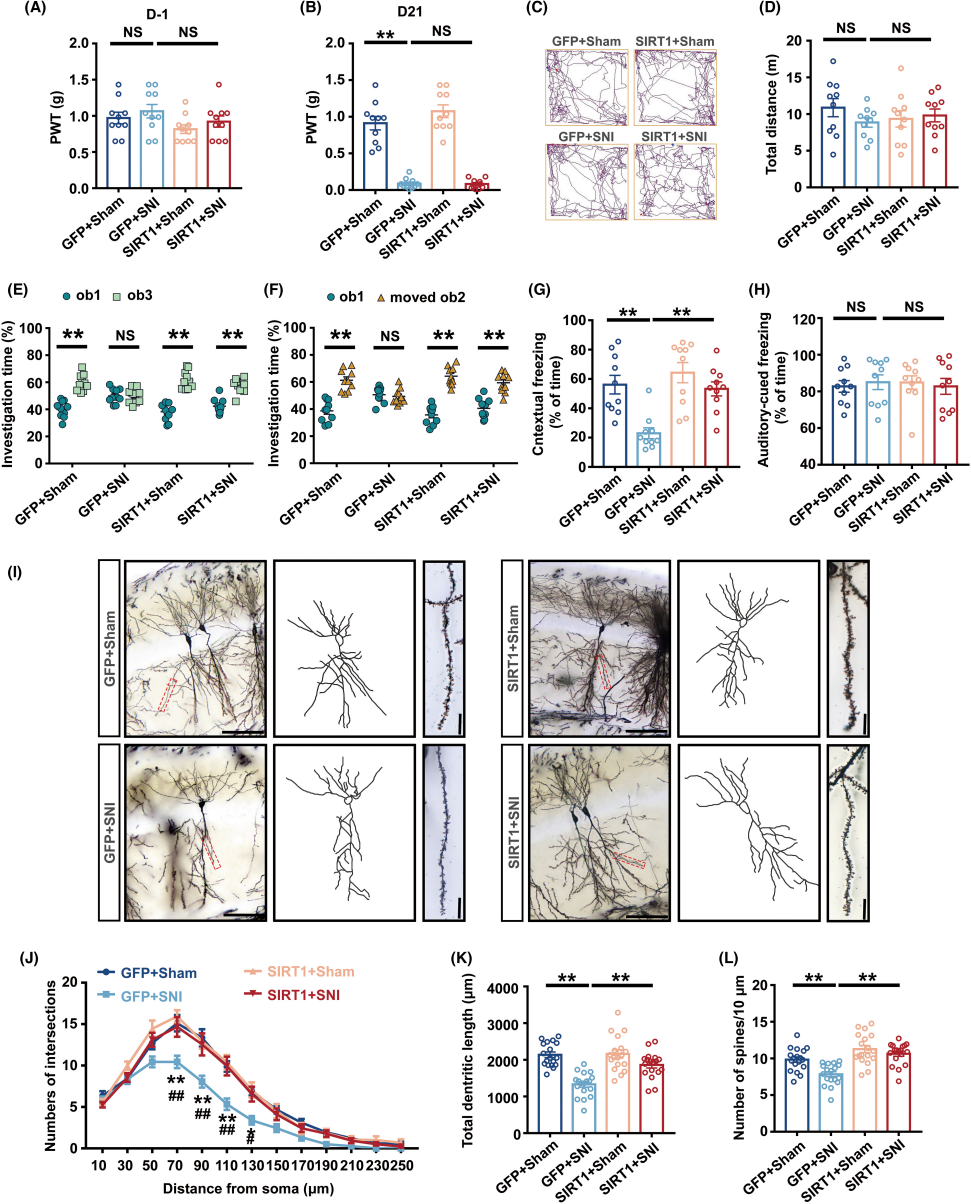
Hippocampal synaptic plasticity injury and cognitive dysfunction in SNI mice were improved by upregulating SIRT1 expression in glutaminergic neurons of the hippocampal CA1 area. (A) PWT 1 day before the operation (*n* = 10). (B) PWT 21 days after the operation (*n* = 10). (C) Representative diagrams of the four groups in the OFT. (D) The total movement distance of the four groups (*n* = 10) in the OFT. (E) Investigation time (%) to novel object in NORT test (*n* = 10). (F) Investigation time (%) to new location in OLT test (*n* = 10). (G) Freezing time in the context test (*n* = 10). (H) Freezing time in the tone test (*n* = 10). (I) A hippocampal profile image of Golgi staining of hippocampal CA1 neurons, 20× with camera tracings, and 60× for spine counting. Scale bar, 100 μm for 20×; 10 μm for 60×. (J) Quantitation of dendritic intersections (*n* = 18). **p < 0.01, *p < 0.05 versus the GFP + Sham group; ^##^ *p* < 0.01 versus the SIRT1 + SNI group. (K) Quantitation of the total dendritic length (*n* = 18). (L) Quantitation of the dendritic spine density. **p < 0.01; NS, no significance. Error bars indicate SEM. SIRT1, silent information regulator 1; PWT; paw withdrawal threshold; OFT, open field test; FCT, fear conditioning test.

The AAV‐SIRT1 vector was locally microinjected into the hippocampal CA1 area of C57 mice to specifically overexpress SIRT1, and the AAV‐GFP virus was injected as a control to determine the role of overexpressing SIRT1 in glutaminergic neurons on the cognitive dysfunction caused by chronic pain (Figure 5A). Confocal imaging of virus‐injected brain sections revealed that the virus was only locally expressed in the hippocampal CA1 area (Figure 5B). Western blotting was performed to examine protein expression in the hippocampus. SNI modeling‐induced SIRT1 reduction was reversed by the overexpression virus (Figure 5C,D). Additionally, immunofluorescence analysis showed that the overexpression virus increased the rate of colabeling between SIRT1 and glutaminergic neurons and reversed the effects of SNI on SIRT1 expression in glutaminergic neurons (Figure 5E,F). Overexpression of SIRT1 in glutaminergic neurons of the hippocampal CA1 area did not enhance the PWT in SNI mice (Figure 6A,B). Additionally, the mice's capacity for exercise was unaffected by the overexpression virus (Figure 6C,D). In the NORT or OLT experiments, the SIRT1+SNI group mice showed a significant increase in the percentage of investigation time for new object or object with new position compared to familiar ob1 exploration, whereas there is no significant difference for the exploring time between familiar ob1 and novel ob3, or between familiar ob1 and moved ob2 in GFP+SNI group mice (Figure S4A,B and Figure 6E,F). Moreover, during the FCT training phase, no significant difference was observed among the four groups in freezing time (Figure S4C). The overexpression virus increased the freezing time in the context test, reversing the SNI‐caused loss of hippocampus‐dependent context fear memory but having no impact on hippocampus‐independent tone fear memory (Figure 6G,H).

Golgi‐cox labeling was conducted to find out the changes of the synaptic plasticity of neurons. With the overexpression of SIRT1, the number of dendritic branches between 70 and 130 μm from the cell body center, the overall length of the dendrites, and the density of the dendritic spines in SNI mice all increased (Figure 6I–L). What's more, SIRT1 overexpression significantly reduced the synaptic gap and increased the thickness of PSD in SNI mice by TEM detection (Figure S4D–F). Hippocampal PSD95 expression (Figure S4G,H) and long‐term potentiation (Figure S4I,J) were also markedly enhanced by overexpression of SIRT1 in CA1 glutaminergic neurons of SNI mice.

## 4 DISCUSSION

**FIGURE 7:**
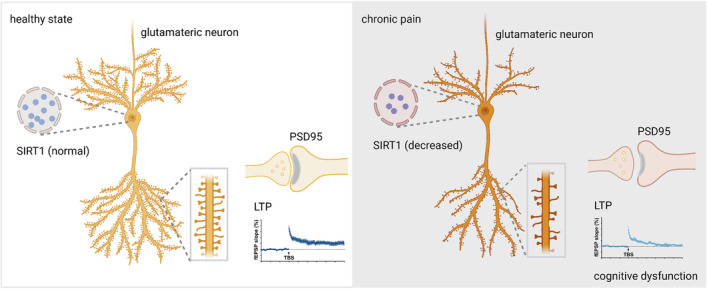
The diagram illustrates the role of SIRT1 in the development of chronic pain‐related cognitive dysfunction. After nerve injury, the reduction in SIRT1 levels leads to fewer dendritic crossings, shorter overall dendritic length, decreased dendritic spine density, lower PSD95 levels, reduced thickness of the postsynaptic densities, widened synaptic fissure, and lower fEPSP slope, which ultimately results in chronic pain‐related cognitive dysfunction.

Our findings suggested that the context fear memory impairment observed in the FCT of SNI mice may be connected to changes in synaptic plasticity caused by the downregulating SIRT1 expression in glutaminergic neurons (non‐GABAergic neurons) of the hippocampal CA1 area (Figure 7). Selective knockdown of SIRT1 in glutaminergic neurons in the hippocampal CA1 region of healthy mice could cause synaptic plasticity damage and induce cognitive dysfunction in mice. In SNI mice, overexpression of SIRT1 in glutamatergic neurons in the hippocampal CA1 region or increased SIRT1 enzyme activity could significantly improve the harm to hippocampal synaptic plasticity and the cognitive dysfunction caused by chronic pain. In conclusion, this research reveals that SIRT1 is a crucial regulatory protein in the emergence of cognitive impairment caused by chronic pain. By reducing synaptic plasticity injury, SIRT1 in glutaminergic neurons of the hippocampal CA1 area may ameliorate cognitive impairment caused by chronic pain.

A growing body of research has demonstrated that chronic pain might hinder the development of hippocampus‐dependent memory.^30^, ^34^, ^35^, ^36^, ^37^, ^38^ Recent work has shown that these cognitive functions—perception, imagination and recall of scenes and events—all engage the anterior hippocampus.^39^ However, there are few studies on context‐based fear memory in chronic pain states. According to our research, SNI mice had a shorter freezing time in the context test 24 h after training, but hippocampus‐independent tone fear memory was unaffected another 24 h later. This finding supports earlier research findings that prolonged pain affects the development of hippocampus‐related context fear memory.^40^, ^41^ Moreover, SNI mice exhibited the same freezing time as Sham mice during the training phase, which indicated that the two groups had the same capacity for fear conditioning learning and that chronic pain may influence the storage but not the formation of fear memory.

Synapses are crucial components of neuronal connections. The alteration in the number and makeup of synapses during plasticity is one of the primary processes regulating synaptic strength.^42^ Rapid synaptic plasticity is necessary for memory development and maintenance in the hippocampus.^43^, ^44^ An increasing number of studies indicate that impairment of hippocampal synaptic plasticity in neurodegenerative diseases such as Alzheimer's disease (AD) and Parkinson's disease (PD) has been proven to be associated with cognitive decline.^12^, ^45^, ^46^, ^47^, ^48^, ^49^, ^50^ Short‐chain fatty acid supplementation improves aberrant synaptic transmission in the hippocampal CA1 region, consequently reducing cognitive impairment caused by prolonged postoperative pain.^51^ Moreover, many studies confirmed that the hippocampal CA1 region plays a crucial role in contextual fear memory.^14^, ^52^, ^53^, ^54^, ^55^ Therefore, we analyzed the hippocampal synaptic structure changes in the hippocampal CA1 region of SNI mice by detecting the synaptic areas and synaptic ultrastructure. Our research revealed that the number of synapses, the total length of dendrites, and the number of dendritic branches were decreased in SNI mice. Previous studies have shown that PSD is increased due to the increase in PSD95, which helps to increase the early structure of dendritic spines.^56^, ^57^ Ultrastructural damage to hippocampal synapses in mice with diet‐induced cognitive impairment has been confirmed.^28^ In our study, the excitatory synaptic gap widened, and the thickness of the postsynaptic dense substance decreased with the expression levels of PSD95 decreased, which indicated that SNI mice had abnormal synaptic ultrastructure. In hippocampal sections prepared from partial ligation of the sciatic nerve model mice, long‐term potentiation was maintained at a significantly lower level than that in sham‐treated mice.^58^ Amitriptyline enhanced cognitive function and increased long‐term potentiation in the right hippocampus in chronic migraine rats.^16^ We found that SNI mice showed a reduced ability to induce and maintain high‐frequency stimulation of Schaffer collaterals, indicating impaired synaptic functional plasticity. These findings suggest that both structural and functional synaptic plasticity in the hippocampal CA1 region of SNI mice with cognitive dysfunction were impaired.

SIRT1 is crucial for cognitive function, and low levels are abnormally associated with the cognitive impairment of neurodegenerative disorders.^17^, ^18^, ^19^, ^20^, ^59^ Enriching the environment, resveratrol, and other treatments to increase SIRT1 levels or activate SIRT1 can ameliorate cognitive impairment caused by aging, stress, or ischemia.^60^, ^61^, ^62^ In addition, activation of SIRT1 enhances, whereas its loss‐of‐function impairs, dendritic branching, axon development, and axon extension.^20^, ^63^, ^64^ Reduced SIRT1 by repeated neonatal propofol exposure plays an important role in cognitive dysfunction by suppressing synaptic plasticity.^32^ Besides, in cases of neurotoxicity, SIRT1 may also play an important protective function.^65^, ^66^ SIRT1 expression or enzyme activity in the ventral tegmentum, amygdala, and dorsal root ganglion is reduced in chronic pain model mice. However, the changes in SIRT1 expression in the hippocampus of mice with chronic pain are still unclear. Previous studies have shown that hippocampus is crucial for formation and maintenance of cognitive dysfunction.^67^ Therefore, in this study, we evaluated the expression changes of SIRT1 in the hippocampus and examined the neuronal specificity of the changes. Our findings demonstrated that SNI caused a downregulation in SIRT1 expression in glutaminergic neurons of the hippocampal CA1 area. To verify the exact role of SIRT1 in synaptic plasticity and cognitive function, we regulated SIRT1 expression and enzyme activity by injecting viral vectors or drugs into the hippocampal CA1 region. We discovered that selective knockdown of SIRT1 in glutaminergic neurons of healthy mice reduced synaptic regions by decreasing the total length of dendrites, the number of dendritic branches, and the number of synapses and caused cognitive dysfunction. These findings suggest that SIRT1 in glutaminergic neurons of the hippocampal CA1 region is a crucial protein molecule for the development and maintenance of cognitive dysfunction and synaptic plasticity. In addition, hippocampal synaptic plasticity was enhanced when the hippocampal CA1 region of SNI mice was injected with AAV‐SIRT1 or SIRT1720. Specifically, in the hippocampus of SNI mice, overexpression of SIRT1 or increased SIRT1 enzyme activity increased the total length of dendrites, the number of dendritic branches, and the density of dendritic spines. At the same time, the cognitive dysfunction of SNI mice was alleviated.

Our experimental results also found that knocking down and overexpressing SIRT1 in hippocampal CA1 glutaminergic neurons, as well as SRT1720 injection in the hippocampal CA1 region, did not significantly affect the paw withdrawal threshold in mice. This finding suggests that SIRT1 in hippocampal glutaminergic neurons may not have a direct impact on the sensory components of pain in mice but may be an important molecule mediating chronic pain‐induced cognitive impairment. Its exact role and mechanism still need further exploration. It was reported that activation of PKA/SIRT1 signaling pathway by photobiomodulation therapy improved memory and cognitive ability in a mouse model of AD.^17^ Antiallergic drug desloratadine (DLT) stimulated autophagy process and repressed neuroinflammation through 5HT2AR/cAMP/PKA/CREB/Sirt1 pathway.^68^ It might be a promising therapeutic strategy for AD. According to the studies mentioned above, the PKA/SIRT1 pathway is crucial for cognition, which may also be a potential mechanism under chronic pain related cognitive dysfunction. Previous studies have shown that brain‐derived neurotrophic factor (BDNF) regulates the growth of dendrites and the morphology of dendritic spines by regulating actin and microtubule synaptic proteins in dendrites, thereby regulating synaptic structural plasticity.^57^, ^69^ In addition, BDNF promotes the release of the neurotransmitter glutamate before the synapse and increases the number of AMPA‐type glutamate receptors (AMPARs) at the postsynaptic membrane, increasing synaptic transmission intensity or efficiency.^70^ A recent study suggested that SIRT1 mediates reversible deacetylation of MeCP2 binding at the BDNF promoter in the hippocampus.^71^ In addition, SIRT1 normally downregulates the expression of miR‐134 via a repressor complex containing the transcription factor YY1, and unchecked miR‐134 expression following SIRT1 deficiency results in the downregulated expression of CREB and BDNF.^20^ Thus, the regulation role of SIRT1 on BDNF via different signaling pathways may be a potential mechanism by which SIRT1 improves cognitive impairment associated with chronic pain, which need to be further researched.

## 5 CONCLUSIONS

Hippocampal synaptic plasticity injury mediated by SIRT1 downregulation is involved in chronic pain‐related cognitive dysfunction. Upregulating and activating SIRT1 may alleviate cognitive dysfunction of SNI mice by improving synaptic plasticity. These data provide novel insight into understanding the pathogenesis of chronic pain‐related cognitive dysfunction and identify a potential therapeutic target.

## CONFLICT OF INTEREST STATEMENT

The authors declare no conflict of interests.

## Supporting information


Data S1.

Figure S1.

Figure S2.

Figure S3.

Figure S4.

## Data Availability

The data that support the findings of this study are available from the corresponding author upon reasonable request.
