# Supplementary material for: Hippocampal synaptic plasticity injury mediated by SIRT1 downregulation is involved in chronic pain‐related cognitive dysfunction
Source: CNS Neurosci Ther. 2023 Aug 17;30(2):e14410. doi: 10.1111/cns.14410 (PMC10848102; doi:10.1111/cns.14410)

The lanes of the unedited gel/blot that appear in the cropped image in the manuscript have been highlighted in red box.

Full unedited gel/blot for Figure 1K

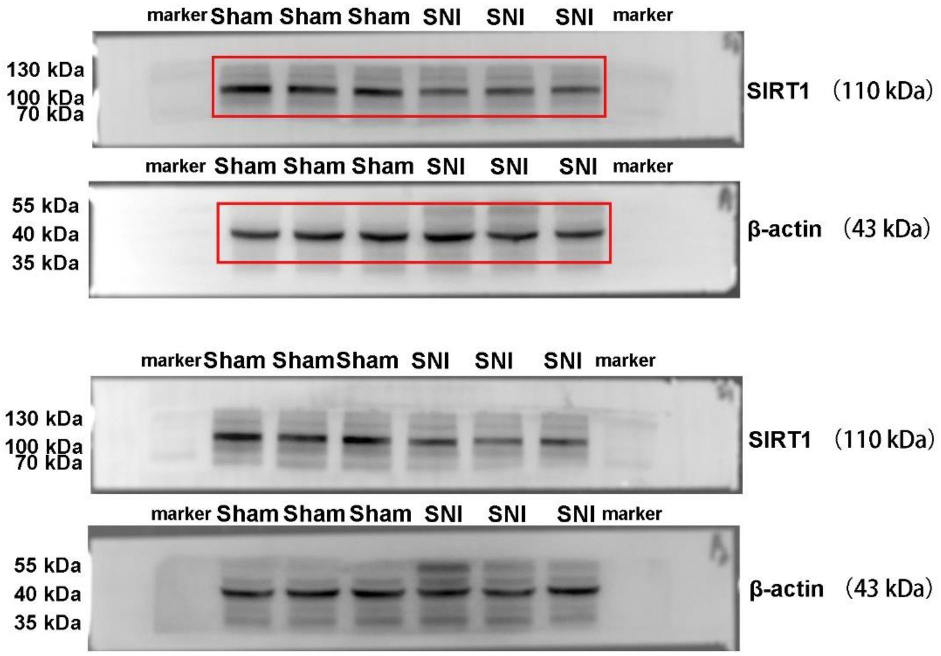

Full unedited gel/blot for Figure 2D

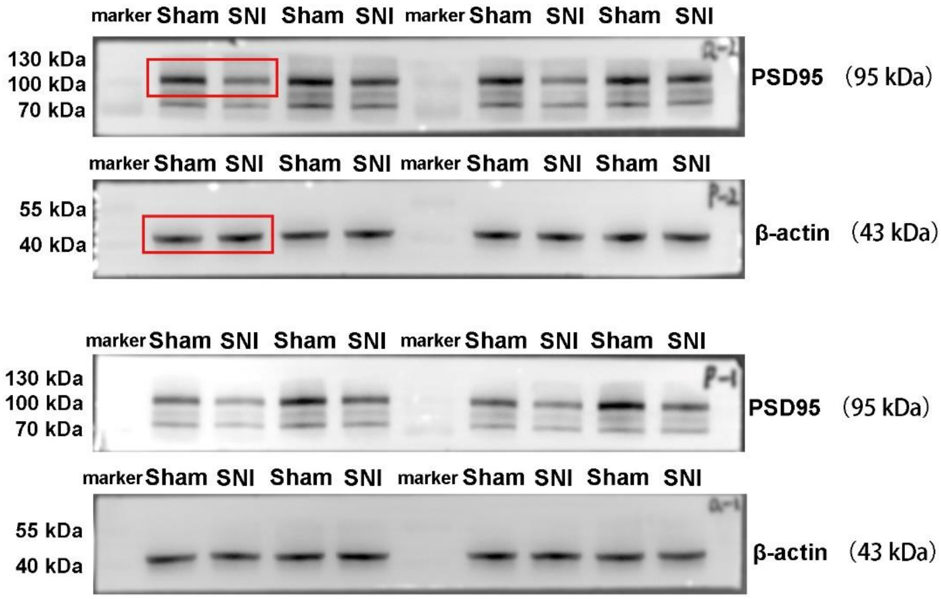

Full unedited gel/blot for Figure 3C

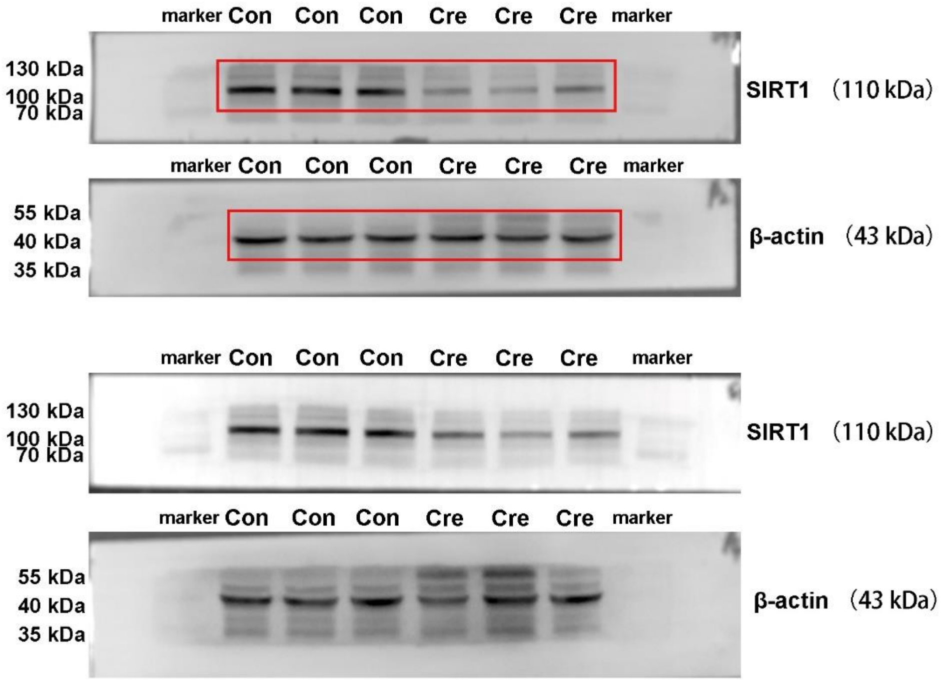

Full unedited gel/blot for Figure 4C

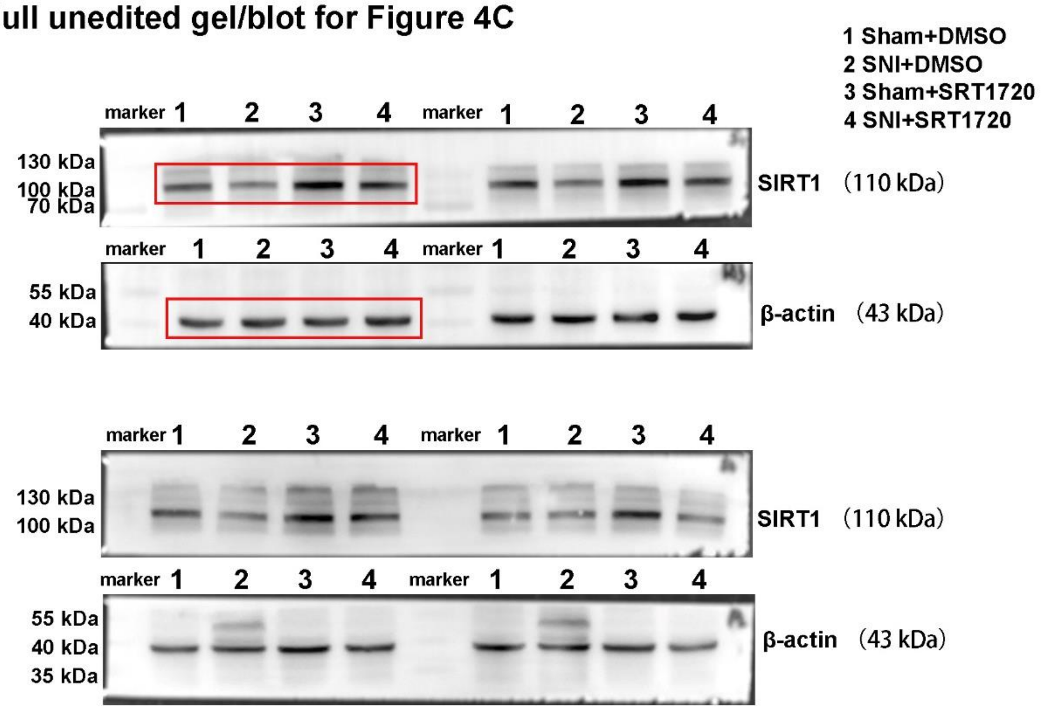

Full unedited gel/blot for Figure 5C

1 GFP+Sham
2 GFP+SNI
3 SIRT1+Sham
4 SIRT1+SNI

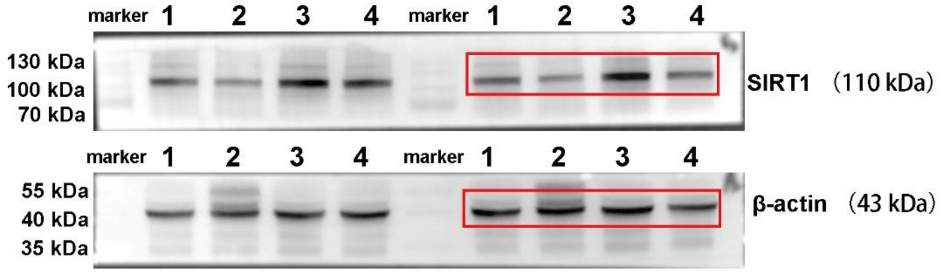

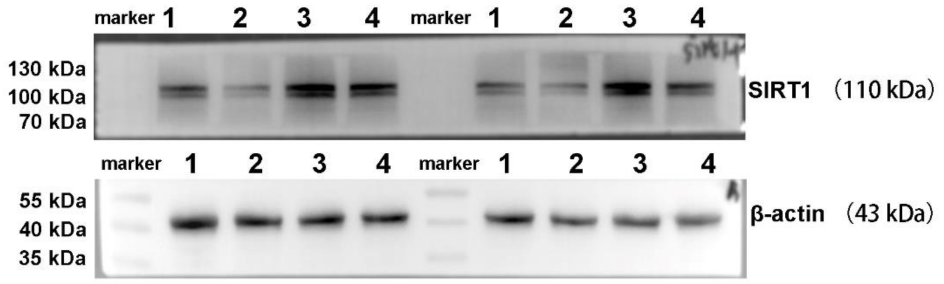

Full unedited gel/blot for Figure S2G

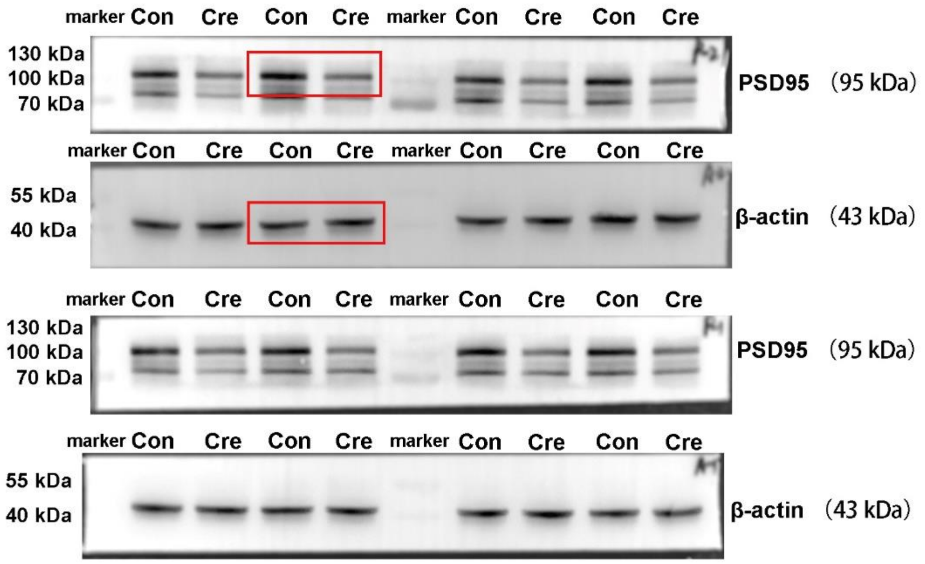

Full unedited gel/blot for Figure S3G

1 Sham+DMSO
2 SNI+DMSO
3 Sham+SRT1720
4 SNI+SRT1720

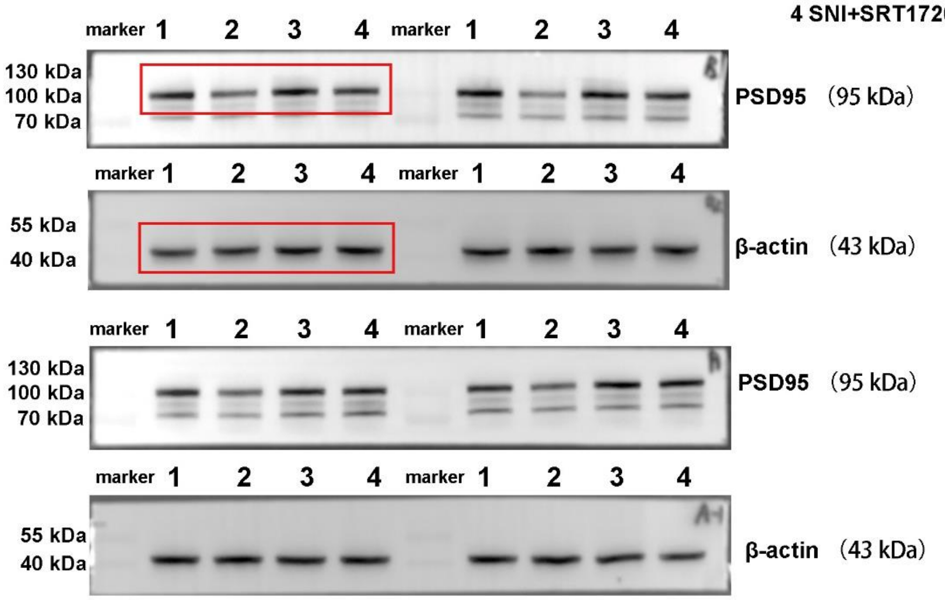

Full unedited gel/blot for Figure S4G

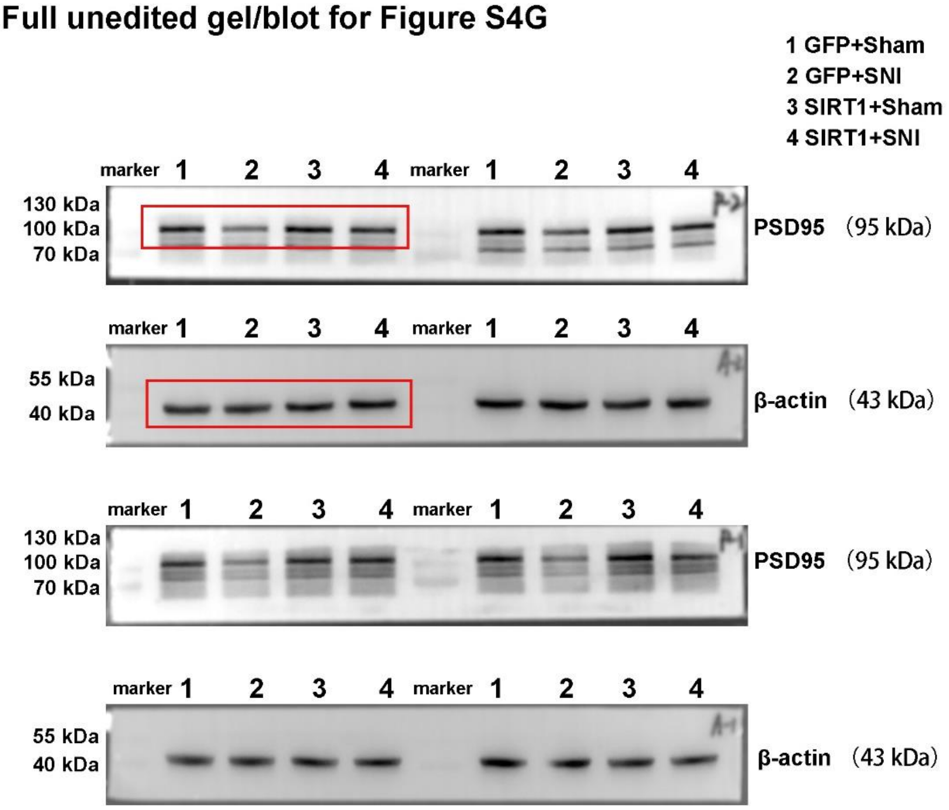

Supplement: Supplementary file 1 — Data S1. [file CNS-30-e14410-s002.pdf]
