# Supplementary figures and images for: Hippocampal synaptic plasticity injury mediated by SIRT1 downregulation is involved in chronic pain‐related cognitive dysfunction
Source: CNS Neurosci Ther. 2023 Aug 17;30(2):e14410. doi: 10.1111/cns.14410 (PMC10848102; doi:10.1111/cns.14410)

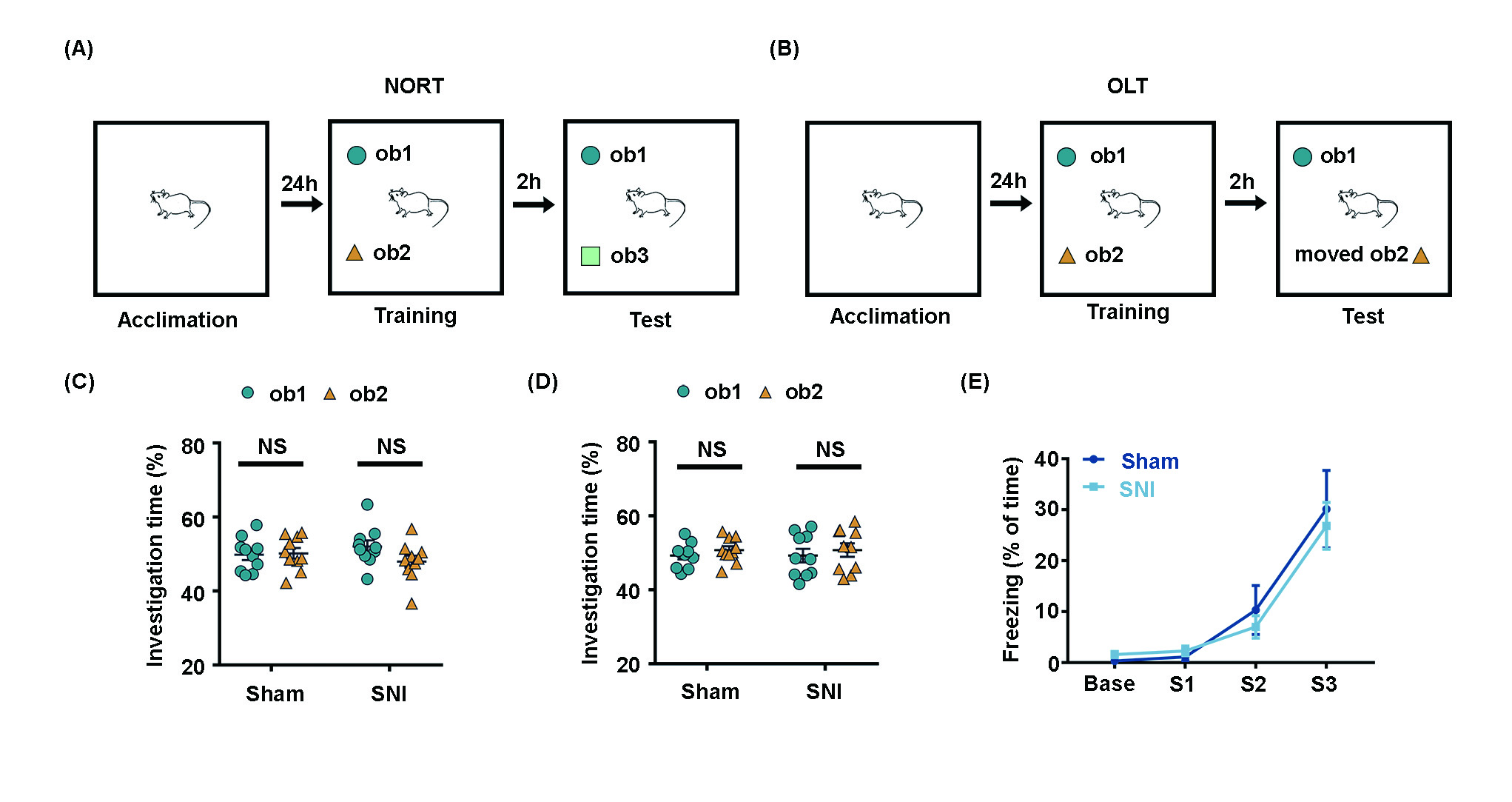

Supplement: Supplementary file 2 — Figure S1. [file CNS-30-e14410-s005.jpg]

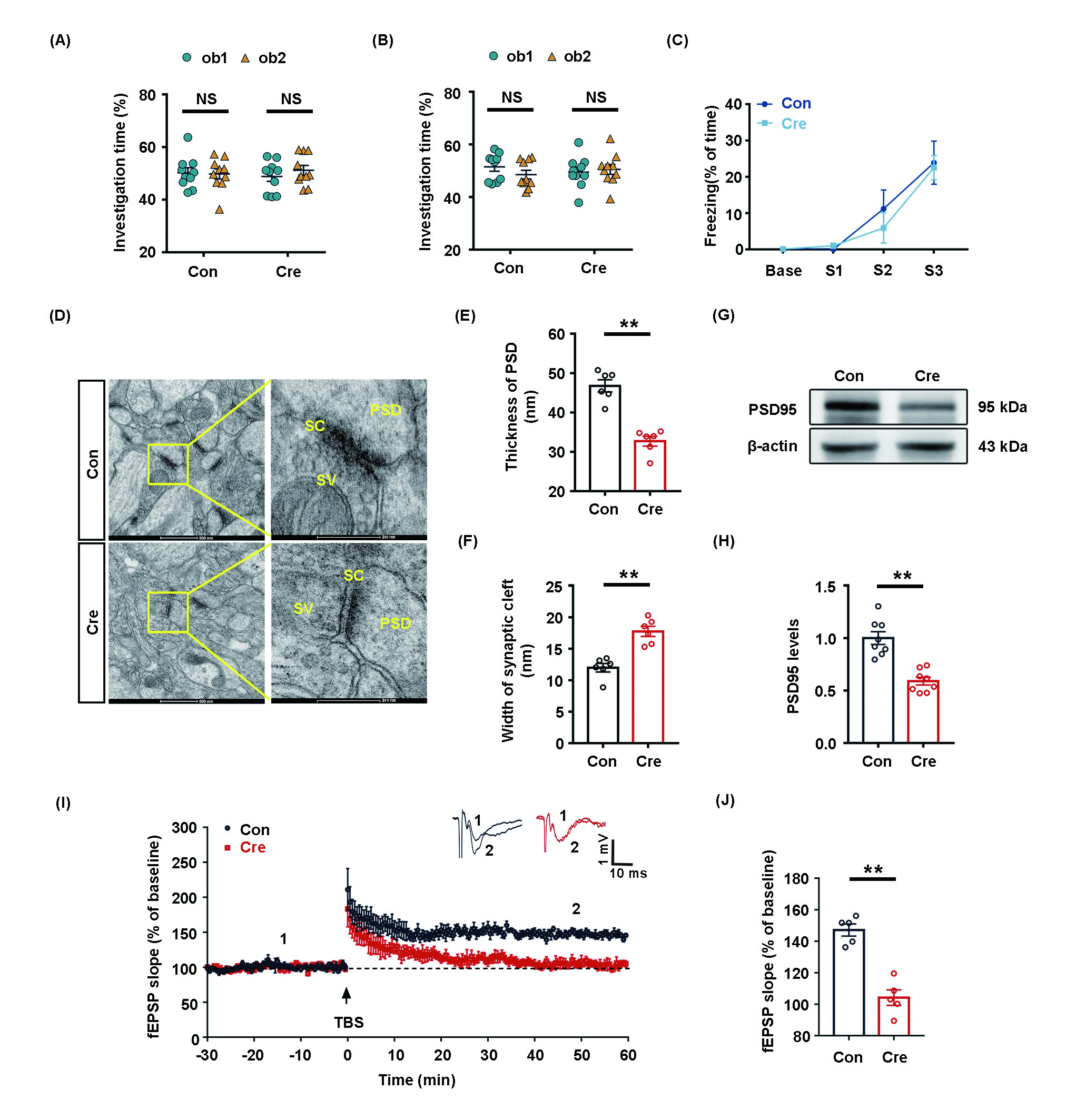

Supplement: Supplementary file 3 — Figure S2. [file CNS-30-e14410-s001.jpg]

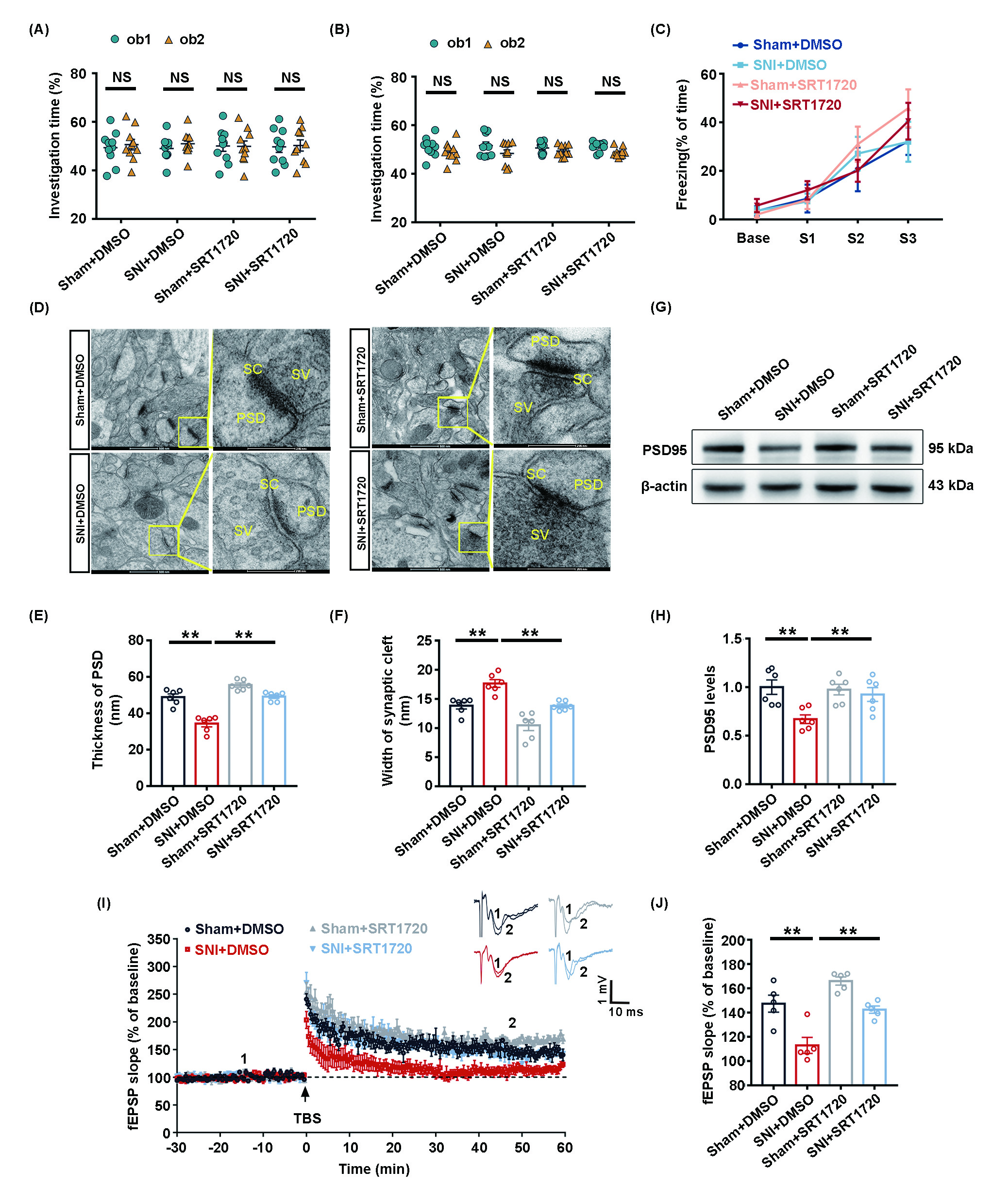

Supplement: Supplementary file 4 — Figure S3. [file CNS-30-e14410-s003.jpg]

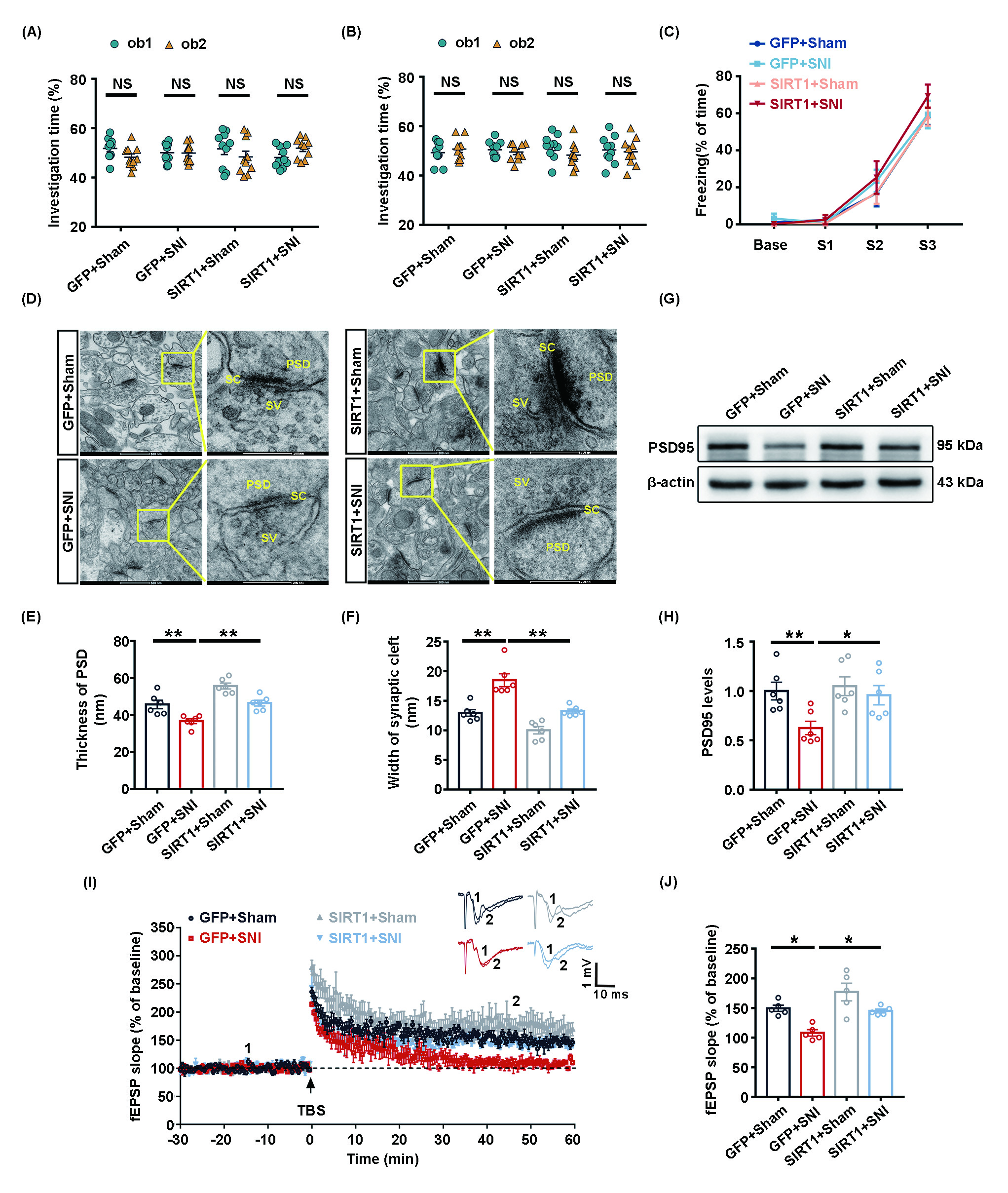

Supplement: Supplementary file 5 — Figure S4. [file CNS-30-e14410-s004.jpg]
